# Examining transcranial random noise stimulation as an add-on treatment for persistent symptoms in schizophrenia (STIM’Zo): a study protocol for a multicentre, double-blind, randomized sham-controlled clinical trial

**DOI:** 10.1186/s13063-021-05928-9

**Published:** 2021-12-28

**Authors:** Jerome Brunelin, Marine Mondino, Julie Haesebaert, Jerome Attal, Michel Benoit, Marie Chupin, Sonia Dollfus, Wissam El-Hage, Filipe Galvao, Renaud Jardri, Pierre Michel Llorca, Laurent Magaud, Marion Plaze, Anne Marie Schott-Pethelaz, Marie-Françoise Suaud-Chagny, David Szekely, Eric Fakra, Emmanuel Poulet

**Affiliations:** 1 Centre Hospitalier Le Vinatier, PSYR2 team, Bat 416 – 1st floor; 95 boulevard Pinel, 69678, F-69500 Bron cedex, France; 2 INSERM, U1028; CNRS, UMR5292; Lyon Neuroscience Research Center, PSYR2 Team, F-69000 Lyon, France; 3 Lyon 1 University, F-69000 Villeurbanne, France; 4 Université Jean Monnet Saint Etienne, F-42000 Saint Etienne, France; 5 Hospices Civils de Lyon, Pôle Santé Publique, Service Recherche et Epidémiologie Cliniques, F-69003 Lyon, France; 6 Research on Healthcare Performance RESHAPE, INSERM U1290, Université Claude Bernard Lyon 1, Villeurbanne, France; 7 CHU Montpellier, F-34295 Montpellier, France; 8 CHU Nice, F-06001 Nice, France; 9 Paris Brain Institute – Institut du Cerveau (ICM), Inserm U 1127, CNRS UMR 7225, Sorbonne Université, F-75013 Paris, France; 10 CATI Multicenter Neuroimaging Platform, F-75000 Paris, France; 11 CHU Caen, F-14033 Caen, France; 12 CHRU de Tours, CIC 1415, INSERM, Tours; UMR 1253, iBrain, Université de Tours, INSERM, F-37044 Tours, France; 13 University in Lille, INSERM U1172, CHU Lille, Lille Neuroscience & Cognition Research Centre, Plasticity & SubjectivitY (PSY) team, CURE Platform, Lille, France; 14 CHU de Clermont-Ferrand, F-63003 Clermont-Ferrand, France; 15 GHU PARIS Psychiatrie & Neurosciences, site Sainte-Anne, Service Hospitalo-Universitaire, F-75014 Paris, France; 16 Université de Paris, F-75005 Paris, France; 17 CH Princess Grace, MC-98000 Monaco, Monaco; 18 CHU de Saint Etienne, F-42000 Saint Etienne, France; 19 Psychiatric emergency service, Hospices civils de Lyon, F-69005 Lyon, France

**Keywords:** Schizophrenia, Noninvasive brain stimulation, tDCS, tRNS, Hallucination, Negative symptoms

## Abstract

**Background:**

One out of three patients with schizophrenia failed to respond adequately to antipsychotics and continue to experience debilitating symptoms such as auditory hallucinations and negative symptoms. The development of additional therapeutic approaches for these persistent symptoms constitutes a major goal for patients. Here, we develop a randomized-controlled trial testing the efficacy of high-frequency transcranial random noise stimulation (hf-tRNS) for the treatment of resistant/persistent symptoms of schizophrenia in patients with various profiles of symptoms, cognitive deficits and illness duration. We also aim to investigate the biological and cognitive effects of hf-tRNS and to identify the predictors of clinical response.

**Methods:**

In a randomized, double-blind, 2-arm parallel-group, controlled, multicentre study, 144 patients with schizophrenia and persistent symptoms despite the prescription of at least one antipsychotic treatment will be randomly allocated to receive either active (*n* = 72) or sham (*n* = 72) hf-tRNS. hf-tRNS (100–500 Hz) will be delivered for 20 min with a current intensity of 2 mA and a 1-mA offset twice a day on 5 consecutive weekdays. The anode will be placed over the left dorsolateral prefrontal cortex and the cathode over the left temporoparietal junction. Patients’ symptoms will be assessed prior to hf-tRNS (baseline), after the 10 sessions, and at 1-, 3- and 6-month follow-up. The primary outcome will be the number of responders defined as a reduction of at least 25% from the baseline scores on the Positive and Negative Syndrome Scale (PANSS) after the 10 sessions. Secondary outcomes will include brain activity and connectivity, source monitoring performances, social cognition, other clinical (including auditory hallucinations) and biological variables, and attitude toward treatment.

**Discussion:**

The results of this trial will constitute a first step toward establishing the usefulness of hf-tRNS in schizophrenia whatever the stage of the illness and the level of treatment resistance. We hypothesize a long-lasting effect of active hf-tRNS on the severity of schizophrenia symptoms as compared to sham. This trial will also have implications for the use of hf-tRNS as a preventive intervention of relapse in patients with schizophrenia.

**Trial registration:**

ClinicalTrials.gov NCT02744989. Prospectively registered on 20 April 2016

## Administrative information

**Table:** 

Title {1}	Examining transcranial random noise stimulation as an add-on treatment for persistent symptoms in schizophrenia (STIM’Zo): a study protocol for a multicentre, double-blind, randomized sham-controlled clinical trial
Trial registration {2a and 2b}.	Clinicaltrials.gov registration number: NCT 02744989, first posted on April 20, 2016
Protocol version {3}	version 13 on July 13th, 2021, substantial modification n°10
Funding {4}	The French Ministry of health, DGOS, PHRC 14-0042 (2014)
Author details {5a}	1. Centre Hospitalier Le Vinatier, PSYR2 team, Bat 416 – 1st floor; 95 boulevard Pinel, 69678, F-69500, Bron cedex, France 2. INSERM, U1028; CNRS, UMR5292; Lyon Neuroscience Research Center, PSYR2 Team, F-69000, Lyon, France 3. Lyon 1 University, F-69000, Villeurbanne, France 4. Université Jean Monnet Saint Etienne, F-42000, Saint Etienne, France 5. Hospices Civils de Lyon, Pôle Santé Publique, Service Recherche et Epidémiologie Cliniques, F-69003, Lyon, France 6. Research on Healthcare Performance RESHAPE, INSERM U1290, Université Claude Bernard Lyon 1, Villeurbanne, France 7. CHU Montpellier, F-34295, Montpellier, France 8. CHU Nice, F-06001, Nice, France 9. Paris Brain Institute – Institut du Cerveau (ICM), Inserm U 1127, CNRS UMR 7225, Sorbonne Université, F-75013, Paris,France 10. CATI Multicenter Neuroimaging Platform, F-75000, Paris, France 11. CHU Caen, F-14033, Caen, France 12. CHRU de Tours, CIC 1415, INSERM, Tours; UMR 1253, iBrain, Université de Tours, INSERM, F-37044, Tours, France 13. University in Lille, INSERM U1172, CHU Lille, Lille Neuroscience & Cognition Research Centre, Plasticity & SubjectivitY(PSY) team, CURE Platform, Lille, France 14. CHU de Clermont-Ferrand, F-63003, Clermont-Ferrand, France 15. GHU PARIS Psychiatrie & Neurosciences, site Sainte-Anne, Service Hospitalo-Universitaire, F-75014, Paris, France 16. Université de Paris, F-75005, Paris, France 17. CH Princess Grace, MC-98000, Monaco, Monaco 18. CHU de Saint Etienne, F-42000, Saint Etienne, France 19. Psychiatric emergency service, Hospices civils de Lyon, F-69005, Lyon, France
Name and contact information for the trial sponsor {5b}	Aleandre PACHOT, Deputy Director of Clinical Research and Innovation Hospices Civils de Lyon, Direction de la Recherche Clinique et de l’Innovation, Siège Administratif, BP 2251, 3 Quai des Célestins, 69229 LYON Cedex 02 Tél : 0033 472 406 852, Fax : 0033 472 406 869 Alexandre.pachot@chu-lyon.fr
Role of sponsor {5c}	The study sponsor had no role in the design of this study and no any role during its execution, analyses, interpretation of the data, or decision to submit results. The funder of the study had no role in study design, data collection, data interpretation, or writing of the report.

## Introduction

### Background and rationale {6a}

Schizophrenia is one of the most disabling and devastating illnesses of the human brain with a prevalence estimated as being between 0.8 and 1.2% of the population, regardless of culture or country [1]. Clinical expression of the illness is heterogeneous, and symptoms can be classically classified into five main dimensions: positive, negative, disorganized, anxiety/depression and grandiosity/excitement. The positive dimension includes auditory verbal hallucinations, which constitute one of the most troublesome and frequent symptoms of schizophrenia. Approximately 50 to 80% of patients experience these symptoms [2]. The negative dimension includes emotional blunting, alogia, avolition, anhedonia and social withdrawal [3]. These symptoms have major consequences in terms of functional handicaps [4, 5] and are responsible for a high social cost [6].

Despite some advances in psychopharmacology, up to 30% of individuals with schizophrenia still report auditory hallucinations and negative symptoms, even during treatment with adequate dose and duration of antipsychotic medication [7]. These treatment-resistant/persistent symptoms are associated with significant distress, poor social integration and a negative impact on quality of life [7]. They are also associated with a high risk of full-blown relapse and an increased number of hospitalizations, leading to a poor prognosis and expensive medico-economic impacts. Therefore, the development of alternative approaches to alleviate these treatment-resistant/persistent symptoms represents a major challenge.

Among the innovative potential nonpharmacological treatments, noninvasive brain stimulation methods such as repetitive transcranial magnetic stimulation (rTMS) and transcranial direct current stimulation (tDCS) have been developed in an effort to alleviate treatment-resistant symptoms in schizophrenia. The use of these techniques mostly lies on noninvasive brain stimulation capacity to modulate brain activity and on neuroimaging evidence of abnormal brain activity underlying schizophrenia symptoms. Namely, neuroimaging studies have demonstrated on the one hand a hyperactivity of the left temporoparietal junction while the patient experiences auditory hallucinations [8], and on the other hand, a close relationship between the prefrontal cortex integrity and the severity of negative symptoms [9]. Noninvasive brain stimulation studies have shown that targeting the left temporoparietal junction with inhibitory low-frequency rTMS alleviates auditory hallucinations, and targeting the dorsolateral prefrontal cortex with high-frequency rTMS reduces negative symptoms of SCH [10]. Studies using tDCS with a left frontotemporal electrode montage with the anode over the left dorsolateral prefrontal cortex and the cathode over the left temporoparietal junction showed significant effects on auditory hallucinations [11] (for a review, see [12]). Also, using the same electrode montage, a recent large randomized controlled trial (RCT) including 100 participants also demonstrated a significant effect of tDCS for negative symptoms of schizophrenia [13]. However, in spite of these promising results, some other studies failed to demonstrate any superiority of active rTMS or active tDCS over sham to decrease symptoms of schizophrenia (e.g. [14, 15]) claiming for further large RCT and for the need of optimizing stimulation parameters.

In this line, we propose to modify the shape of the delivered current and to deliver high-frequency random noise stimulation (hf-tRNS) to increase the beneficial clinical outcomes. This is based on numerous studies where hf-tRNS has shown an advantage over other electrical stimulation techniques in boosting perceptual and motor learning [16–22], modulating cortical excitability [23], enhancing working memory [24] and at the clinical level reducing symptoms of tinnitus in patients [25]. Moreover, tRNS also show an advantage in clinical studies for reducing pain in multiple sclerosis [26], for decreasing motor cortex excitability in Parkinson’s disease [27], for reducing depressive symptoms in patients with major depression [28] and, through case reports, alleviating negative symptoms [29] and auditory hallucinations [30] in patients with schizophrenia [31].

Therefore, we propose to develop a large multicentre (13 centres), randomized, controlled trial to investigate the efficacy of frontotemporal hf-tRNS in patients with schizophrenia presenting with persistent disabling symptoms despite the prescription of at least one antipsychotic medication. We will assess the changes in the biological and cognitive outcomes as well as clinical outcomes after the intervention and at several follow-up time points during 6 months. We believe that an in-depth understanding of the biological and cognitive effects of hf-tRNS will constitute an important step toward improving the technique and developing treatment response markers. We will also investigate whether specific profiles/characteristics of patients would be associated with different clinical, cognitive and neural effects.

### Objectives {7}

#### Main objective and primary outcome

Objective 1 is to investigate the acute clinical effect of active hf-tRNS on symptoms of schizophrenia after 10 sessions delivered during 5 consecutive days. We will assess at day 5 the number of responders, defined as patients demonstrating a reduction of at least 25% from baseline on the positive and negative syndrome scale (PANSS [32], a standardized clinical scale evaluating schizophrenia symptoms). We will test the superiority of active hf-tRNS versus sham hf-tRNS to achieve a clinical response.

### Secondary objectives

#### Clinical efficacy

Objective 2a is to investigate the long-term clinical effects of active hf-tRNS on symptoms of schizophrenia compared to sham, measured as the number of responders at 1-, 3- and 6-month follow-up.

Objective 2b is to investigate the acute and long-term effects of hf-tRNS on different clinical aspects of schizophrenia: the five main dimensions of symptoms measured by the PANSS (positive, negative, depression, disorganization and grandiosity/excitement) and other symptoms measured with specific psychometric scales: auditory hallucinations, negative symptoms, depression and quality of life at 1-, 3- and 6-month follow-up.

Objective 2c is to investigate the acute effects of hf-tRNS (at day 5) on source monitoring capacities, a cognitive function that is associated with psychotic symptoms of schizophrenia [33].

#### Response predictors

Objective 3a is to investigate the effects of hf-tRNS according to several socio-demographic and clinical variables. We will assess the effects of age, illness duration, level of treatment failure and symptoms severity on clinical response (i.e. > 25% reduction on the PANSS).

Objective 3b is to investigate the neural markers, i.e. markers of brain anatomy, activity and connectivity, which might influence the response to hf-tRNS as well as to investigate the effects of hf-tRNS on brain activity and connectivity. Brain anatomy, activity and connectivity will be measured using multimodal brain imaging including anatomical magnetic resonance imaging (MRI), diffusion tensor imaging (DTI), resting-state functional MRI and perfusion (arterial spin labelling (ASL)).

#### Attitudes toward treatment

Objective 4a is to investigate the effect of hf-tRNS on the attitudes toward treatment for schizophrenia.

Objective 4b is to assess the attitudes toward hf-tRNS before and after hf-tRNS (active or sham).

Objective 4c is to investigate the relationship between the attitudes toward treatment (before and after hf-tRNS) and clinical variables (adherence, insight, quality of life, nicotine dependence).

#### Aim of two ancillary studies nested in the trial

##### Ancillary 1: Predictive biological marker of response—evaluate BDNF isoform proportion

The objective is to assess whether BDNF isoform proportions at baseline would predict hf-tRNS clinical response at the endpoint. To achieve our aim, the relative proportion of serum brain-derived neurotrophic factor (BDNF) isoforms before hf-tRNS (biomarker study collecting blood samples) will be measured at the baseline visit and compared between a group of future remitters and a group of future non-remitters.

##### Ancillary 2: Social cognition marker—evaluate the effects of hf-tRNS on facial affect recognition (FAR) in patients with schizophrenia with persistent symptoms

In this ancillary study, the secondary objectives are to evaluate the influence of a single session of active hf-tRNS on FAR in patients with schizophrenia with persistent symptoms, in comparison with sham stimulation, and to evaluate whether changes in FAR after a single session would predict the symptomatic improvement after hf-tRNS. FAR will be assessed 3 times, one time at baseline, one time after the first and another after the 10th session of hf-tRNS. The results will be compared between the active and sham groups. A cumulative effect of hf-tRNS sessions on FAR will be assessed by comparing the performances between baseline, post 1 and post 10 sessions.

### Trial design {8}

The study is designed as a pivotal superiority, randomized, double-blind, multicentre, parallel-group, sham-controlled clinical trial. A total of 144 patients with schizophrenia will be randomly assigned to receive either active hf-tRNS or sham in a one-to-one ratio, stratified by study site using an internet platform (IWRS system).

## Methods: participants, interventions and outcomes

### Study setting {9}

The trial is a multicentre study planning to include 13 study sites: academic hospitals and university hospitals in France and one in Monaco (see details in Table 1). Only 11 centres actually recruited participants, and two withdrew from the study due to stimulation devices not complying with the technical requirements of the protocol.

**Table 1:** List of sites of investigation initially planned and recruitment status

Centre no.	City	Hospital	Recruitment status
1	Lyon	*University hospital*	Recruiting
*2*	Nice	*University hospital*	Recruiting
*3*	Lille	*University hospital*	Recruiting
*4*	Tours	*University hospital*	Recruiting
*5*	Clermont-Ferrand	*University hospital*	Recruiting
*6*	St. Etienne	*University hospital*	Recruiting
*7*	Bron	*Psychiatric hospital*	Recruiting
*8*	Paris Sainte Anne	*Psychiatric hospital*	Recruiting
*9*	Monaco	*Hospital*	Recruiting
*10*	Paris Saint Antoine	*Hospital*	Withdrawn
*11*	Caen	*University hospital*	Recruiting
*12*	Strasbourg	*University hospital*	Withdrawn
*13*	Montpellier	*University hospital*	Recruiting

Two ancillary studies on subsets of participants are planned and nested in the trial:

1. A study collecting blood samples to measure a marker of neural plasticity, the BDNF, all the centres are involved (ancillary 1)
2. A study measuring facial affect recognition (FAR), only for 3 centres (Bron, Lyon and Saint Etienne) (ancillary 2).

No stratification will be done at the randomization process to include participants in the ancillary studies. Of note, only 9 of the investigation centres will participate in the MRI acquisition part of the study because of technical constraints of the availability of an MRI scan for research purposes in the city.

### Eligibility criteria {10}

The following are the inclusion criteria:

1. Diagnosis of schizophrenia according to the DSM 5.0 criteria.
2. Presence of symptoms despite the optimization of the antipsychotic dosage (based on prescriber’s judgement) for at least 6 weeks *(i.e. a dosage increase cannot be considered due to tolerability issues and/or is judged unlikely to bring sufficient clinical improvement).* This will be operationalized by a minimum negative subscore of 20 and at least one item scoring > 4 or a minimum positive PANSS subscore of 20 with at least one item scoring > 4 (e.g. delusion or hallucination), indicating persistent negative symptoms and/or persistent positive symptoms.

*NB: We did not stipulate a criterion concerning prior treatment with antipsychotics, but these data will be carefully recorded. For instance, we will include patients on clozapine as well as on other antipsychotics and will examine whether the impact of hf-tRNS differs in these two populations*

3. Patient under curatorship/guardianship or not.
4. Age between 18 and 65 years old.
5. Covered by a public health insurance.
6. Understanding the French language.
7. Signed written informed consent.

The following are the exclusion criteria:

8. Other axis I psychiatric conditions including current diagnosis of a major depressive episode (uni- or bi-polar disorder) according to DSM 5

*NB: Patients with substance-related and addictive disorders will not be excluded from the study, but these data will be carefully recorded.*

9. Contraindications for transcranial electrical stimulation (neurologic stimulator, pacemaker, cardiac defibrillator, cardiac prosthesis, vascular prosthesis, intracranial clips or clamps, cerebrospinal fluid derivation, metallic splinters in the eyes)
10. Changes in total composite PANSS score of at least 20% between screening and enrolment visits
11. Pregnancy (controlled by urine pregnancy test in females of childbearing age)
12. Clinical condition requiring inpatient procedure under constraint

Patients likely to be included will be screened by the study investigators blind for the treatment procedure during specialized consultations in academic hospitals, which may take place in outpatient or during hospitalization. A checklist will allow the investigator to screen each of the inclusion and non-inclusion criteria of the approached patients. A psychometric assessment will be carried out by the centre investigator in order to check the inclusion criteria (PANSS score). A list of previous treatments will be made. Treatment stability will be checked, and detailed information regarding the tRNS procedure and treatment will be given to the eligible patients before the collection of their informed consent. All patients will undergo a medical evaluation that will include a physical examination, routine laboratory studies, drug toxicology screening, electrocardiogram and a urine pregnancy test if the subject is a female of childbearing age. Diagnostic assessments will be made using the Diagnostic and Statistical Manual of Mental Disorders (DSM-5.0) Structured Clinical Interview. The diagnosis of schizophrenia and the verification of the absence of co-morbidities likely to exclude the patients will be performed by clinical interview and by a structured interview using the structured M.I.N.I. 6.0 questionnaire.

#### Who will take informed consent? {26a}

Informed consent will be obtained by local investigators (psychiatrists) that are not current medical doctors of included patients. The investigator who obtains the consent will be the blinded rater of clinical assessments throughout the study period.

Patients will be completely and truthfully informed in comprehensible terms, of the objectives and requirements of the study, any risks incurred, necessary monitoring and safety measures, their right to refuse to participate in the study, and the possibility of withdrawing at any time without incurring any penalty or withholding of treatment on the part of the investigator.

All of this information appears on an information and consent form given to the patient. The patient’s free written informed consent will be obtained by the investigator or the physician representing him prior to final inclusion in the study. One copy of the information and consent form signed by the two parties will be given to the patient. The investigator will keep the other copy. For patients under curatorship/guardianship, information procedure and consent will be made with the curator or the tutor. In the second case, the consent must be signed by the tutor after clear information, and a document will be transmitted to the patient and also to the tutor.

For any substantial change to the protocol, regarding the study objectives, its design, the population, the examinations or significant administrative aspects, a new consent will be obtained from the persons participating in the research.

#### Additional consent provisions for collection and use of participant data and biological specimens {26b}

Patients will be asked if they consent to participate in the study and to all the secondary objectives (optional or not) and the ancillary studies including MRI acquisition, blood sampling for biological analysis and FAR task, depending on the centre of the investigation. Separate information sheets are provided for the FAR ancillary study, and separate written informed consent must be signed by the patient after receiving full information. Refusal to participate in the optional or ancillary studies is not limitative for participation in the pivotal STIM’Zo study.

### Interventions

#### Explanation for the choice of comparators {6b}

Sham stimulation is of key importance in transcranial electrical stimulation studies [34]. Using the chosen sham procedure, which has been developed by the manufacturer of the stimulator, will allow sensations to be felt in the scalp, which are equivalent to those of the active stimulation. The same device will be used for both the sham and the active procedures, thus keeping the patients blinded for the treatment conditions. Furthermore, the randomization between active and sham stimulations is made by IWRS. The investigator responsible for delivering the treatment (that is not the investigator in charge of rating the clinical scales) will have a code (given by the sponsor of the study) that will be entered into the device before the session, thereby ensuring the double-blind procedure (thus, the investigator issuing the treatment has no knowledge of the randomization). Moreover, the study being a parallel arm study, the participants will have no means of comparison between the active and sham stimulations. It should be noted that we have already used this sham procedure during randomized studies with parallel arms in patients with schizophrenia, in patients with obsessive-compulsive disorder and in healthy volunteers (e.g. [11, 35, 36]). The participants were unable to significantly guess the sham treatment from the active treatment. We plan to control the safety and the blinding of the procedure using a 12-item visual analogue scale (VAS) evaluating the acute effects of tRNS delivered to the participant each day, at the end of the second daily sessions of hf-tRNS. The blinding of the participant and of the clinical rater will be evaluated at visit number 2 after the 10 hf-tRNS sessions using two VAS evaluating the confidence to guess the condition of stimulation between 0 and 100.

#### Intervention description {11a}

The study intervention consists of 10 sessions of active or sham high-frequency transcranial random-noise stimulation (hf-tRNS) delivered with a transcranial electrical stimulator, an active medical device of class IIa. Systems from two commercial distributors are allowed in the study:

- NeuroConn GmbH, Albert-Einstein-Straße 3, 98693 Ilmenau, Germany (phone: +49 3677 689790; email: info@neuroconn. Reference: DC-Stimulator Plus
- Neuroelectrics – Avda Tibidao 47 bis – 083038 Barcelona, Spain (phone +34 93 254 03 66; email: info@neuroelectrics.com. Reference: StarStim tDCS system

Stimulation will be performed between two 7×5 cm (35 cm^2^) sponge electrodes soaked in a saline solution (0.9% NaCl). The participants will be sitting comfortably in a quiet room. Electrodes will be placed in accordance with the international 10–20 electrode placement system. The anode will be placed with the middle of the electrode over a point midway between F3 and FP1 (left prefrontal cortex: dorsolateral prefrontal cortex, assumed to correspond to a region including Brodmann’s areas [BA] 8, 9, 10 and 46, depending on the patient). The cathode will be located over a point midway between T3 and P3 (left temporo-parietal junction, assumed to correspond to a region including BA 22, 39, 40, 41 and 42, depending on the patient). The stimulation intensity will be set at 2 mA for 20 min during stimulation sessions twice a day for 5 consecutive weekdays. The twice-daily sessions will be separated by at least 2 h. The choice of electrode montage was based on conformity with those for the treatment of schizophrenia symptoms [11, 13]. This electrode montage was also in accordance with studies reporting a beneficial effect of noninvasive brain stimulation on FAR in both healthy volunteers [37–40] and patients with psychiatric disorders [41, 42]. The procedure for patient’s installation and electrode placement will be standardized between study centres using specific training formation sessions and dedicated videos (one video for each stimulator device—Starstim and Neuroconn—was available).

Stimulation parameters were adapted according to the latest data from the literature on noninvasive brain stimulation. The experimental group will receive the ACTIVE stimulation: high-frequency random noise stimulation (100 to 500 Hz), intensity = 2 mA, offset = + 1 mA, session duration = 20 min, ramp up/ramp down = 30 s and total number of sessions = 10 (twice daily sessions separated by at least 2 h for 5 consecutive weekdays).

The control group will receive the SHAM stimulation following the same regimen (i.e. twice daily sessions separated by at least 2 h for 5 consecutive weekdays). Sham stimulation will consist of a 20-min session including 40 s of active stimulation (same parameters as in the ACTIVE arm) at the beginning of the sessions (ramp up/ramp down = 30 s) whatever the stimulator.

#### Criteria for discontinuing or modifying allocated interventions {11b}

Discontinuation or modification of the allocated intervention is not allowed regarding the stimulation sessions. In such cases, the patient will be withdrawn from the study.

#### Strategies to improve adherence to interventions {11c}

No specific strategies were taken to improve adherence to interventions. The only strategy that is planned is to deliver the 10 hf-tRNS sessions over 5 consecutive days instead of a classical transcranial electrical stimulation protocol with once-daily session over 10 working days as described in numerous studies in patients with psychiatric conditions [43]. This may allow decreasing the number of dropouts during the acute treatment phase and allow achieving the primary outcome of the study. Using such a strategy in a previous 3-month study in hallucinating patients with schizophrenia, only a 10% attrition rate was observed.

#### Relevant concomitant care permitted or prohibited during the trial {11d}

During the acute phase, the antipsychotic treatment prescribed at the time of inclusion will remain stable for the full length of the study, i.e. throughout the active phase and up to M+6 of the maintenance phase. The general rule is no dosage or treatment change can be performed with regard to this treatment. Exceptionally, if there is a need to adapt the dose or change the antipsychotic treatment during the maintenance phase of the study:

- If clinical worsening is observed by the study investigator and justifies, according to his judgement, an adjustment of the therapeutic treatment throughout the therapeutic protocol, the patient will be withdrawn from the study.
- If no clinical worsening is observed by the study investigator, the patient remains in the study and must be followed to completion according to protocol.

In both cases, the adjustment of antipsychotic treatment (change or dose) must be recorded in the eCRF.

Adjustments of prescribed complementary anxiolytic treatments will be recorded in the eCRF.

#### Provisions for post-trial care {30}

The participants will remain under double blind during all of the follow-up phase without modification of the antipsychotic treatment (general rules). The clinical and the adverse reaction evaluations will be carried out for a duration of 6 months, as described in the schedule. Patients will be kept blinded to treatment conditions until the end of the pivotal study (planned in 2022).

After inclusion, no simultaneous participation in other interventional clinical research will be authorized during the study. At the end of the study, there will be no exclusion period.

### Outcomes {12}

#### Primary endpoint (linked to the main objective)

Our primary outcome (objective 1) will be the number of responders in the active and the sham group after 10 sessions of hf-tRNS. The clinical response will be defined according to Leucht et al. [44], by a decrease of at least 25% of the value of positive and negative syndrome scale (PANSS) between baseline and after the 10 sessions of hf-tRNS. This will allow us to measure the acute clinical effects of hf-tRNS.

#### Secondary endpoints

##### Clinical efficacy

Outcomes for objective 2a will be the number of responders, as previously defined, at 1, 3 and 6 months after the acute treatment phase. This will allow us to measure the maintenance effect of hf-tRNS.

Outcomes for objective 2b will be as follows:

- Schizophrenia symptoms will be assessed using total PANSS scores and using the dimensional pentagonal analysis described as an example by Lindenmayer et al. [45]. This approach, which will allow the assessment of positive, negative, depression, disorganization grandiosity/excitement dimensional scores and the composition of items included in each of the sub-dimension, will be confirmed by an analysis of the distribution of PANSS scores on our own sample of participants.
- Auditory hallucinations will be assessed using Auditory Hallucination Rating Scale (AHRS), Hallucination Changes Scores (HCS) [46, 47] and the Psycho-Sensory hAllucinations Scale (PSAS) scores [48], a specific multi-modal rating scale that explore hallucinatory modalities and severity.
- Depressive symptoms will be assessed by the Calgary Depression Scale for Schizophrenia (CDSS) [49].
- Sensory gating-like experiences will be assessed by scores at the French Sensory Gating Inventory (SGI) [50], which measures the information on 4 dimensions of sensory gating-like experiences.
- Negative symptoms of schizophrenia will be assessed by BNSS scores, Brief Negative Symptom Scale [51] and Self-evaluation of Negative Symptoms (SNS) scores, the self-evaluation of negative symptoms [52, 53].
- Global symptom severity and treatment response will be assessed by the score at the Clinical Global Impressions Scale (CGI) [54].
- Quality of life will be assessed by the score at the Schizophrenia Quality of Life Questionnaire Short Form (S-QoL18) [55].
- Nicotine dependence will be assessed by the Fagerström Test for Nicotine dependence (FTND) [56].
- These outcomes will be measured at baseline, after the 10 sessions of hf-tRNS, at 1-, 3- and 6-month follow-up, except for the S-QoL18 and the FTND which will only be evaluated at baseline and after 6 months and for the SNS, evaluated at baseline and after 1 and 3 months.

Outcomes for objective 2c will be the number of misattributions (confusion between internally and externally generated thoughts or actions) during a specific source-monitoring task, see the “Description of clinical scales and assessment tools” section [57].

##### Response predictors

Outcomes for objective 3a: The predictive effect of age, clinical characteristics (illness duration, previous treatment failure, nicotine dependence) and baseline scores at clinical scales defined in objective 2b on the clinical outcome (as defined for objective 1) will be assessed.

Outcomes for objective 3b: Brain anatomy, activity and connectivity at baseline and after the 10 sessions of hf-tRNS will be measured. Namely, the outcomes will be the (1) brain anatomy (measured using anatomical magnetic resonance imaging (MRI-T1)), in particular, grey matter volume, cortical thickness and gyrification; (2) structural connectivity (measured using diffusion tensor imaging (DTI)), in particular, the fractional anisotropy; (3) functional connectivity (measured using resting-state fMRI); and (4) perfusion (measured using arterial spin labelling (ASL)).

##### Attitudes toward treatment

Outcomes for objective 4a will be the attitudes toward medication for the treatment of schizophrenia assessed by BMQ scores (Beliefs about Medicines Questionnaire [58–60]) at baseline and 3 months after hf-tRNS.

Outcomes for objective 4b will be attitudes toward transcranial electrical stimulation (tES) assessed by scores at an adapted version of BMQ (BMQ tES) at baseline and at 3-month follow-up.

Outcomes for objective 4c will be the insight into the illness assessed by the Scale to Assess Unawareness of Mental Disorder (SUMD) scores [61, 62] and the adherence to medication measured by the Medication Adherence Rating Scale scores (MARS) [63, 64] at baseline and at 3-month follow-up.

The SUMD, MARS, BMQ and BMQ tES evaluations will be performed 3 months after the last hf-tRNS session.

#### Ancillary studies

1. *Outcome for the BDNF ancillary study* will be the proportion of total and isoforms of BDNF (total BDNF, pro-BDNF and mature BDNF), a marker of neuronal plasticity, in serum obtained before the first hf-tRNS session.
2. *Outcome for the FAR ancillary study* will be the variation of the proportion of correct answers obtained on the FAR test (Ekman computerized test) [65] before stimulation (baseline, D0) and after the 10th sessions of hf-tRNS (D5). The secondary endpoints of the ancillary study will be as follows: the variation of the proportion of correct answers obtained on the FAR test (Ekman computerized test) before stimulation (D0 or D1 before the 1st session) and after a single stimulation session (D1, after the 1st session) and correlation between, on one hand, the variation of the proportion of correct answers obtained on the FAR test before stimulation (D0) and after a stimulation session (D1) and, on the other hand, the variation of the total PANSS score before stimulation (D0) and after stimulation (D5).

#### Participant timeline {13}

The schedule of enrolment, interventions, assessments and visits for participants is illustrated in Table 2.

**Table 2:** Participant timeline

	Baseline		Acute phase	Follow-up period			
	V0	V1		V2	V3	V4	V5
	D-30 to D-7	D-7 to D0	D1 to D5	D5 to D10	D 35 ± 3	D 90 ± 7	D 180 ± 7
Screening eligibility, demography, medical and psychiatric^a^ history, treatment, pregnancy test^b^	X						
Written informed consent and enrolment		X					
PANSS evaluation	X	X		X	X	X	X
Randomization^c^			X (D1)				
Drug toxicology screening and AP		X					
Physical and clinical examination		X		X	X	X	X
hf-tRNS sessions^d^			X				
FTND		X			X		X
SNS		X			X	X	
AHRS, HCS, PSAS, CDSS, CGI		X		X	X	X	X
MRI^e^, SGI, source memory^f^		X		X			
Blood samples^g^			X (D1)				
FAR^h^		X	X (D1)	X			
SQoL-18		X					X
BNSS		X		X			X
MARS, BMQs, SUMD		X				X	
Medication deviation^i^, AE		X	X	X	X	X	X

^a^Length of the episode, number of treatments, number of previous treatments, hospitalizations, symptom severity, disease duration—diagnostic of schizophrenia and the verification of the absence of comorbidities will be performed using the MINI 6.0

^b^Urinary test

^c^Patients will be randomly allocated to receive either active or sham hf-tRNS sessions

^d^hf-tRNS sessions will be delivered twice a day separated by at least 2 h for 5 consecutive weekdays from D1 to D5 (Monday to Friday, 10 sessions)

^e^MRI will include an anatomical MRI sequence, a resting-state fMRI sequence and DTI sequences; an optional perfusion ASL sequence will also be performed in two centres

^f^Source memory task investigating patient’s capacity to distinguish between internally and externally generated words

^g^Collection of 2 × 5 mL in vacutainers of blood between 8 and 9 am in fasting patients will be taken of the first hf-tRNS session (D1) for ancillary study 1

^h^The FAR test will be performed before the 1st session (V1 or D0 or D1 before the first session), between the 1st and the 2nd session of hf-tRNS (D1) and after the 10th session (V2) for ancillary study 2

^i^The medicinal antipsychotic treatments will remain stable for the full length of the study (D1 to D120). The patient will be withdrawn from the study if there is a need to adapt the dose or change the antipsychotic treatment (see exception)

#### Sample size {14}

Based on our previous transcranial electrical stimulation study in patients with schizophrenia [11, 66], we assume that 50% of patients in the active hf-tRNS group and 20% of patients in the sham hf-tRNS group will be responders (> 25% decrease of PANSS scores between D0 and D5). Based on this estimated difference, we calculated that 125 patients are required to demonstrate a difference between the 2 groups with a power of 95% (*p* = 0.05). The attrition rate in our previous study was 15%. Therefore, we assume that the recruitment of 144 patients will be necessary to achieve our goals.

Patients who withdraw from the study before the first hf-tRNS session will be replaced. The criteria for withdrawal from the study are as follows:

- Clinical worsening observed by the study investigator and justifying, according to his judgement, are adjustments of the therapeutic treatment throughout the therapeutic protocol (from D1 to D5).
- Serious adverse reaction for which the responsibility of the study is in question.
- Withdrawal of consent.

The BDNF study is an exploratory study (ancillary study 1); no specific sample size calculation was done to achieve this objective. We expected that a large majority of included patients will allow to participate in this ancillary study. Regarding the FAR ancillary study 2, based on the previous studies showing preliminary findings on the impact of rTMS and tDCS on FAR in schizophrenia [41, 42], we considered a difference in correct answers in the FAR task between the groups (active stimulation versus sham) of 7.3% with a standard deviation of 7.15. With an *α* risk of 5%, a power of 90%, and a bilateral test and taking into account an attrition rate of 10%, we estimate that two groups of 23 participants (46 participants in total) will be required.

### Recruitment {15}

In order to achieve our objectives, we developed a multicentre study with 12 centres in France and 1 in Monaco (10 active centres). All the investigators from these centres have already proven their capacities to achieve noninvasive brain stimulation studies in patients with psychiatric conditions as highlighted by several international publications. All the investigators from these centres are full active members of the French association for the use of noninvasive brain stimulation in psychiatry (see https://www.afpbn.org/sections/step/) that annually organized scientific meetings and intensive courses on the use of noninvasive brain stimulation in psychiatry. Moreover, a majority of the investigators have already participated in a large national study investigating the clinical interest of low-frequency rTMS in patients with major depression (see [67]) and in other multicentric studies in schizophrenia [68]. Annual joint meetings between investigators are planned, and periodical newsletters will be sent to all the study investigators and staff during the study period for updates on the state of the study. In case of difficulties in recruitment, new centres could be open and some centres could be closed.

We have carried out a feasibility study among the participating centres. After verification of the inclusion criteria, 1 patient included every 2 months is realistic. Based on our feasibility study, we estimate that 10% of patients will not be randomizable. According to our hypothesis, each centre will include at least 5 patients per year. An inclusion period of 3 years will be sufficient to include 144 patients. Fewer patients are required to achieve brain imaging goals (Table 3).

**Table 3:** Expected recruitment by centre and by year in the pivotal clinical study and in the neuroimaging outcome part

Centre no.	City	Brain imaging outcome	***n*** expected/year	Total (3 years)
1	Lyon	Yes	5	15
*2*	Nice	Yes	6–7	20
*3*	Lille	Yes	5	15
*4*	Tours	Yes	6	18
*5*	Clermont-Ferrand	Yes	5	15
*6*	St. Etienne	Yes	5	15
*7*	Bron	Yes	5	15
*8*	Paris Saint Anne	Yes	6-7	20
*9*	Monaco	**No**	5	15
*10*	Paris Saint Antoine^a^	**No**	6	18
*11*	Caen	Yes	4	12
*12*	Strasbourg^a^	Yes	2	6
*13*	Montpellier	**No**	3–4	10
	***TOTAL***		**65**	**194**

^a^The two centres withdrew from the study

## Assignment of interventions: allocation

### Sequence generation {16a}

Patients will be randomly assigned to either the active hf-tRNS group (*n* = 72) or the sham hf-tRNS group (*n* = 72) with a one-to-one allocation ratio using an online platform (Interactive Web Response System (IWRS system)). The randomization will be stratified per study site (centre) using block randomization. Block size is not disclosed to ensure concealment. The randomization list will be generated and used by the sponsor of the study without any interaction with investigators and will be unavailable to those who enrol participants or assign interventions.

There is no stratification due to participation in any of the ancillary studies or in the neuroimaging part.

### Concealment mechanism {16b}

Randomization will be performed using an online platform (IWRS system) on the day of the first hf-tRNS session to ensure allocation concealment and avoid the allocation of a sequence that will not be used.

### Implementation {16c}

All patients who give consent for participation and fulfil the inclusion criteria will be enrolled by investigators. They will be assigned a unique anonymous identification code, composed of the number of the study site (centre number), the patient initials (first letter of the name, first letter of the surname) and the last number from 1 to 144. This number will be assigned automatically by an e-CRF. Patients will be randomized by a staff member (investigator or study nurse) using an online platform (IWRS system), resulting in random allocation into one of 2 study arms. The randomization system will provide a 5-digit number code that will be entered into the stimulator device used for delivering hf-tRNS (the code will correspond either to active or sham hf-tRNS). The randomization code will be delivered in the eCRF the day of the first hf-tRNS session.

## Assignment of interventions: blinding

### Who will be blinded {17a}

Blinding will be maintained at 4 levels: trial participants, care providers, outcome assessors and data analysts.

Blinding for the hf-tRNS condition will be achieved for the investigator who will administer the hf-tRNS by the use of a randomization code that will be entered into the stimulator device and for the patients by ensuring identical appearance and sensation for the two hf-tRNS conditions (see details in §6b). Clinical/psychometric assessments, imaging and biological data will be collected and analysed by investigators blind to group assignment and different from the investigator who will administer the hf-tRNS.

### Procedure for unblinding if needed {17b}

The first page of the CRF will explain the unblinding procedure. In case of an emergency, the investigator or the sponsor will have to call a specific phone number at the “poison control centre” from Lyon that is open 7/7. The “poison control centre” is an independent third party, different from the sponsor and the investigator, who will be given the list of randomization codes and the corresponding allocation.

## Data collection and management

### Plans for assessment and collection of outcomes {18a}

All participating investigators have already been trained for the PANSS, AHRS-HCS assessment for a prior trial using a video assessment of patients validated by the scientific committee. In order to control the validity of the assessments and in particular the inter-rater accuracy, all investigators will have to complete one assessment session before beginning inclusions based on a movie from an interview with a patient and to transmit their evaluation to the principal investigator. The principal investigator will compare the results with the validated assessment. In case of more than 10% variation, the investigator will have to complete a second assessment session with another patient. It is important to note that the proposed scales are classical for research in this pathology and that all study sites are experts in the treatment of schizophrenia.

### Description of clinical scales and assessment tools

#### Positive and Negative Syndrome Scale (PANSS)

The PANSS is a 30-item clinician-rated scale of the psychopathological symptoms observed in patients experiencing psychotic states, in particular, schizophrenia [32]. The items are rated from 1 to 7. It allows the calculation of the scores for three syndromic dimensions (positive, negative and general psychopathology), both from a categorical and dimensional perspective. It is particularly recommended for determining a psychopathological profile, to look for the predictive elements of an evolution and to evaluate the respective efficacies of diverse therapeutic strategies. The PANSS measurements will be obtained by observing the patient behaviour during an interview, as well as from the clinical interview and reports from primary care staff or family members. The measurements result in summary scores on a scale of 7 positive items, 7 negative items and 16 general psychopathological items. The PANSS measurements should be based entirely on the details concerning a specific period, normally the previous week. A second analysis based on a pentagonal organisation will also be measured as secondary objectives in the trial.

#### The Mini-International Neuropsychiatric Interview (M.I.N.I. 6.0)

Lifetime MINI questionnaire consists of a structured diagnostic interview, with a short completion time, exploring in a standardized manner the principal 1st axis of psychiatric troubles of the DSM 5.0. In order to eliminate potential false positives, the diagnostic validity of the results from this interview will be controlled during a clinical interview at visit inclusion. The MINI is divided into modules that correspond to a diagnostic category. At the beginning of each module, one or more screening questions corresponding to the main criteria of the trouble are presented. At the end of each module, one or more diagnostic boxes allow the clinician to indicate if the diagnostic criteria have been met.

#### Auditory Hallucinations Rating Scale (AHRS) and Hallucination Change Score (HCS)

The AHRS is a clinician-rated outcome measure of auditory hallucinations severity, which allows the measurement of 7 dimensions (frequency, loudness, number of voices, real nature of the voices, importance of the content, behavioural modification and the demanding nature of the voices) within a 24-h period. The assessment of changes between the baseline evaluation, and the follow-up evaluation will be completed by a single item evaluation of auditory hallucination changes, the Hallucination Change Score (HCS). Initially validated by Hoffman et al. [46], this tool has been translated and validated in French by our team [47]. It is used in the majority of work testing the effect of noninvasive brain stimulation on auditory hallucinations.

#### Psycho-Sensory hAllucinations Scale (PSAS)

The PSAS [48] is a clinician-rated scale assessing the severity of hallucinations across several sensorial modalities. It includes auditory, visual, gustatory and olfactory and coenesthetic hallucinations. Each domain was declined in the same way: a descriptive part that uses questions to sort for the presence or absence of hallucinations, and a qualitative and a quantitative part. Initially conceptualized to assess hallucination in both patients with schizophrenia and patients with Parkinson’s disease, a supplementary “guardian angel” domain, defined by this feeling of presence to the vivid sensation that somebody is present nearby, when no one is actually there, in the absence of sensory clues revealing a presence, is also scored. This structured interview is designed to elicit specific details regarding different hallucinations. When asking questions, the interview is designed to rate the patient’s experiences over the last 7 days except for “frequency” in the description part. Moreover, in this part, “Duration”, “perception”, “unpleasant or negative aspects”, “conviction”, “impact” and “control” should be rated according to the widely prevalent episode in the last 7 days. The assessment of auditory hallucinations will be particularly followed along with this study (change from baseline); however, this scale can also access switches between hallucination modes.

#### Clinical Global Impression (CGI)

The CGI involves the on-off assessment of the severity of the illness, the assessment of overall improvement and the measurement of the therapeutic index. Item 1 will be completed during the initial assessment. Items 2 and 3 will be filled-in during the assessments that follow.

The disease severity item is a good overall measure of the patient’s current condition and gives a clear idea of the patient’s condition at different visits. The overall impression of improvement allows the evolution of the patient’s condition to be observed. The therapeutic index allows the effect of the medicine to be measured by describing the level of therapeutic efficacy and the side effects.

#### Calgary Depression Scale for Schizophrenia (CDSS)

The CDSS [49] is a 9-item clinician-rated scale that was specifically developed to assess the level of depression in schizophrenia. All ratings of the items are defined according to operational criteria from 0 to 3 during a goal-directed interview. It distinguishes depressive symptoms from negative positive and extrapyramidal symptoms. It has been extensively evaluated in both relapsed and remitted patients and appears sensitive to change. This scale will be used to control for an effect of hf-tRNS on depression since anodal transcranial electrical stimulation has proven efficacy on this symptom, especially in patients with major depressive disorder.

#### Sensory Gating Inventory (SGI)

The SGI is a self-reported questionnaire composed of 36 items addressing a broad range of sensory gating-like subjective experiences that are rated by the patients on a 6-point Likert scale [50]. The psychometric properties of the SGI indicate that it provides valuable information on 4 dimensions of sensory gating-like experiences: perceptual modulation (linked to 16 items, e.g. “My hearing is so sensitive that ordinary sounds become uncomfortable”), over-inclusion (7 items, e.g. “I notice background noises more than other people”), distractibility (8 items, e.g. “There are times when I cannot concentrate with even the slightest sounds going on”) and fatigue-stress modulation (5 items, e.g. “It seems that sounds are more intense when I’m stressed”).

#### Brief Negative Symptom Scale (BNSS)

The BNSS is composed of 13 items organized into 6 subscales anhedonia, distress, asociality, avolition, blunted affect and alogia [51] for the assessment of negative symptoms of schizophrenia. A manual defines the terms used in the scale, provides anchors for each item and gives instructions for a semi-structured interview, including suggested questions. All the items are rated on a 7-point (0–6) scale, with anchor points generally ranging from the symptom’s being absent (0) to severe (6). A scale total score is calculated by summing the 13 individual items; subscale scores are calculated by summing the individual items within each subscale. The BNSS has possible total scores ranging from 0 to 78. The scale was designed primarily for use in treatment trials but may have other applications such as clinical evaluation and tracking of change. The scale does not define a negative symptom subtype.

#### Self-Evaluation of Negative Symptoms (SNS)

The SNS is a French self-administered questionnaire composed of 20 items organized into 5 domains of negative symptoms of schizophrenia (social withdrawal, diminished emotional range, alogia, avolition, anhedonia) presenting 5 subscores comprising the sum of 4 items each [52]. The items are verbatim and noted from 0 to 2: scoring 2 for strongly agree, 1 for somewhat agree and 0 for strongly disagree. The total score is the sum of the 20 items, ranging from 0 (no negative symptoms) to 40 (severe negative symptoms). Patients generally completed the questionnaire in less than 5 min.

#### Quality of life evaluation (S-QoL18)

The S-QoL18 scale is a French self-administered questionnaire measuring the quality of life of the patients [55]. It assesses eight dimensions: psychological well-being, self-esteem, family relationships, relationships with friends, resilience, physical well-being, autonomy and sentimental life. The S-Qol-18 is a short quality of life instrument that has a high degree of comparability with S-QoL-41 [69] and presents satisfactory psychometric properties for patients with schizophrenia.

#### Fagerström test for nicotine dependence (FTND)

The FTND is a short form that includes six questions designed to estimate the degree of nicotine dependence in tobacco smoking. Responses are added to compute a score ranging from 0 (least dependent smokers) to 10 (most dependent) [56].

### Source memory test

In the source memory tasks, participants had to distinguish between 8 silent imagined-hearing, 8 heard and 8 new non-presented words as described in our previous studies [57]. Words were current French words extracted from a verbal fluency task with the same emotional valence and the same length. During the test, words were presented for a duration of 3 s on a computer screen preceded by an instruction. Each word had its own instruction, i.e. “hear this word”, or “imagine hearing this word”. Immediately after the end of the test, a response grid including the 16 presented words plus 8 new non-presented words (“distractors”, range 0–8) was given to the patient. For each word, participants had to report if the word was externally generated or internally generated, or if the word had not appeared on the screen.

#### Brain imaging

Brain imaging will be acquired only in the centre offering technical resources and expertise on MRI and belonging to the CATI network. Imaging data acquisition protocols will be standardized. Each centre will communicate the imaging data to the Centre for Acquisition and Image Processing (CATI) through a secure web platform (Imagys sustem from the KEOSYS company) which allows pseudonymization and secure transfer.

The CATI is a French national platform first created as part of the Alzheimer. Within this project, the CATI will take care of the standardization of MRI sequences between centres, and the raw data quality control before analyses. The PSYR2 team from the Lyon Neuroscience Research Center (INSERM, U1028, CNRS, UMR5292, Université de Lyon) will be in charge of imaging data analyses.

The MRI sequences will include, for anatomical measurements, a sagittal 3D T1-weighted sequence that will be acquired in about 5 min. The standardized parameters will ensure good contrast and whole-brain coverage with 1-mm isotropic resolution. For the DTI analyses, 2D axial spin-echo echo-planar imaging (EPI) sequences will be acquired in about 20 min. Four T2-weighted images with no diffusion weighting (*b* = 0 s/mm^2^ images) and 60 diffusion-weighted images (*b* = 1500 s/mm^2^) will be acquired with whole-brain coverage and 2-mm isotropic voxels. A fieldmap will be acquired to correct for geometrical distortions induced by the EPI sequence. For functional measurements through changes in BOLD T2-weighted signals, a 2D axial gradient echo EPI sequence will be acquired in about 10 min. The standardized parameters will ensure maximum brain coverage with 3 mm isotropic voxels and 250 repetitions for ensuring statistical significance could be reached during the following analyses. Participants will be instructed to keep their eyes close fixed during the functional acquisition, to refrain from thinking about a precise issue and from sleeping. Only Bron, Lyon and St. Etienne sites will acquire ASL sequences.

Imaging data will be preprocessed and analysed according to the most recent standard procedures in the field available at the time of analysis.

#### Beliefs about the Medicines Questionnaire (BMQ) and BMQ-tES

The BMQ is an 18 items self-questionnaire [58]. Ten items measure the specific beliefs about the prescribed treatment, in terms of its perceived necessity (specific-necessity) and concern about it (specific-concern). A further 8 items assess the general beliefs about medicine, including the perception of harm (general-harm) and overuse (general-overuse). Higher values denote stronger or positive beliefs. The BMQ is a scale adapted to assess beliefs about all medicines for a particular condition as schizophrenia. The French version has been validated among diabetes patients [59] and among schizophrenic patients [60].

The BMQ tES is based on the ten items assessing the specific beliefs from the original scale where the word “medication” has been changed by “tES”. The BMQ tES allows the specific assessment of the beliefs of patients toward tES.

#### Scale to assess unawareness of mental disorder (SUMD)

The SUMD is a semi-structured interview based on a dimensional and quantitative approach of insight. Different forms of insight are assessed (global insight into mental illness, insight into symptoms and insight into symptom aetiology). The SUMD has proven to be a reliable and valid instrument to assess insight into schizophrenia [61]. The French translation of the SUMD has been validated [62]. Several studies have already reported a beneficial effect of noninvasive brain stimulation on insight into the illness [70, 71].

#### Medication Adherence Rating Scale (MARS)

The MARS is a ten-item yes/no self-report instrument. It was developed from two existing scales (the Drug Attitudes Inventory and the Medication Adherence Questionnaire) with the aim of developing a more reliable and valid tool for assessing medication adherence behaviour in psychosis [63]. Validation of the French version of the MARS has been performed [64].

### Ancillary studies

#### Biological measurements for the BDNF study

Blood samples will be taken by a nurse between 8 and 9 am in fasting patients before the first hf-tRNS session. 2 × 5 ml of blood will be collected in a vacutainer, and then left for 20 min at room temperature before being centrifuged (3500*g*) for 20 min at 4 °C. The serum will then be removed (200 μL of serum into 1 ml NUNC tubes), and 8 aliquots will be frozen at ≤ − 24 °C until analyses. Aliquot will be centralized at the PI centre (CH Le Vinatier, pharmacy) before central analysis. Total serum BDNF and BDNF isoforms dosages will be performed in collaboration with the Lyon Neuroscience Research Center (CRNL).

#### *Measurements for the FAR study:* the FAR Ekman test

The Ekman facial affect recognition is based on the validated Ekman pictures database. There are two types of tasks in FAR, the affects identification and the affects discrimination. Identification tasks are the most frequently used because they are more sensitive than discrimination tasks. In the absence of factorial studies of social cognition tests in schizophrenia to guide groupings of measures, we have relied on conventions in the literature. With regard to the most recent meta-analyses, which combine different measures of the identification and discrimination of facial affects, there is a presumed similarity between the existing tasks of FAR. The test relies on the black and white photographs from the validated Ekman database with 8 different identities (4 men and 4 women). Six emotions are presented among the following: happy, sad, anger, surprise, disgust and fear. It includes morphing at 20%, 40%, 50%, 60% and 80%. The images are presented in the following way: fixation cross for 500 ms then stimulus for 500 ms and forced choice between the 6 emotions with a response via the keyboard (2000 ms). There are a total of 240 stimuli for a total turnaround time (including installation and explanation of the instruction) of 20 min.

The design of the current task was conceived in collaboration between Lyon, Saint-Etienne and the University of Geneva and based on a previously developed task in our lab that was widely used across several neuropsychiatric populations [72]. It was validated on a population of healthy volunteers and in patients with major depression, receiving tDCS (see ClinicalTrials.gov identifier NCT02793258). Its feasibility has been tested with a smaller sample of patients suffering from schizophrenia.

### Plans to promote participant retention and complete follow-up {18b}

In order to promote participant retention and honour the participants’ time during the study, participants are able to earn up to 180 euros to cover their travel expenses if they complete all the study visits.

To minimize loss to follow-up, all participants’ data collection visits will be scheduled by the research assistant and reminded to the participants by phone calls the day before. The research assistant will also remind participants of the importance of their participation at each visit. Missed visits will be rescheduled and followed up. Missing a visit did not exclude the participant from the study if the patients can attempt the next planned visit.

### Data management {19}

In each study site, part-time CRAs will deal with the local monitoring of the study (management of appointments, recording and archiving of the data and being a link between the investigators of the study sites). They will ensure the data entry for the study sites into the eCRF for centralization. The eCRF was specifically developed for the need of the study using the “ClinInfo” software. The eCRF is accessible by CRA and investigators on a web-accessible platform with personalized and unique login and passwords (www.clininfoservcies.com). A classical CRF on paper is also available to ensure a direct rating of scale during the interview and to keep secure the self-rated questionnaires that will be given to the participants at each visit.

The methodological coordinator, a part-time associate scientist based in the Public Health Department, will ensure methodological support throughout the project, by principally liaising with the coordinating CRAs and the principal investigator. They will develop the database, as well as the quality control tools for the data. At the end of the study, they will ensure the cleaning and freezing of the database, and then they will proceed with the analysis. Two levels of controls of data are planned, one first level by CRA from the sponsor on site who will compare raw data from medical records and data entered in the eCRF. The second level of control with the quality department will also recheck the eCRF and send queries to the investigators in case of abnormalities according to range check for data values.

According to the guidelines for Good Clinical Practices, the study monitor has to check the eCRF entries against the source documents. The informed consent form will include a statement by which the patients allow the sponsor’s duly authorized personnel (trial monitoring team) to have direct access to original medical records which supports data on the eCRF (e.g. patient’s medical file, appointment books, original laboratory records, etc.). These personnel, bound by professional secrecy, will not disclose any personal identity or personal medical information (according to confidentiality rules).

### Confidentiality {27}

The electronic database will be declared and kept according to the criteria required by the CNIL (National Data Privacy Commission). Paper CRF with self-rated questionnaires given to the participants throughout the study period will be kept on sites for 15 years.

In all study-related documents, the patient references will appear only in the form of an ID code composed of the research site number, the patient’s monogram, and the patient serial number at the research. Persons who have direct access will take all necessary precautions in view of ensuring the confidentiality of information relative to investigational medications, trials and human subjects and in particular with respect to their identity and the results obtained.

### Plans for collection, laboratory evaluation and storage of biological specimens for genetic or molecular analysis in this trial/future use {33}

Aliquots from blood samples for ancillary BDNF study will be centralized at the PI site, Pharmacy, CH Le Vinatier, Bron. Samples will be kept frozen for storage at − 24 °C until dosages. Samples from all the participants from all the centres of investigation will be analysed on the same day to avoid any external variation using enzyme-linked immunosorbent assay (ELISA).

## Statistical methods

### Statistical methods for primary and secondary outcomes {20a}

In general, all demographic and clinical characteristics of patients at baseline and all endpoints will be summarized using descriptive statistics and graphs as appropriate. The continuous variables will be summarized as the mean and standard deviation for the normal distributions, median and range for the others. The qualitative variables will be summarized as the effectiveness and the percentage for each method.

Statistical tests used for between-group comparisons will be performed two-sided at a significance level set at 5%, unless otherwise specified.

#### Patient characteristics at inclusion

A bivariate analysis will check for the absence of an initial imbalance of these characteristics between the randomly selected groups.

#### Handling of withdrawals from the study and missing data

The withdrawals from the study will be described as follows: arm in which the patient was assigned, date of withdrawal from the study, reason, characteristics at inclusion and the last values assessed for the patient. The analyses will be based on the intention-to-treat set, including all randomized patients with at least one post-tRNS score (10 sessions completed). Missing data will be replaced with the “last observation carried forward” method until the endpoint. A per-protocol analysis is also planned.

### Analysis of efficacy

#### Primary endpoint

The primary endpoint is the number and rate of responders in each treatment group defined as the ratio of the number of responders on the total number of patients in the group after the 10 sessions of hf-tRNS. Response to treatment is defined as a reduction of at least 25% from baseline at the PANSS. The proportion of responders will be estimated with a 95% confidence interval (95%CI) according to the binomial distribution and compared using a chi-square test. A binomial test will be used to test the superiority of the proportion of responders to hf-tRNS treatment in the active group compared to the placebo group. This analysis can be adjusted or stratified by the study centre.

#### Secondary endpoints analysis

##### Objective 2a

- Responder rates (decrease as mentioned above) at 1 month, 3 months and 6 months will be compared between the groups using an overall chi-square test.
- PANSS scores, at baseline, 5 days, 1 month, 3 months and 6 months, will be compared between the groups using the Wilcoxon rank test.
- The analysis of the evolution of the clinical effects of active treatment versus sham will quantify and test the effect of the intervention and the effect of the treatment group on the values and the slope of change in PANSS score before intervention and after 5 days, 1 month, 3 months and 6 months. To account for the correlation of the score from the same patient and the same group, we will use a mixed linear regression model with a random effect on the intercept or general average. The effect of the intervention (before/after) and the effect of the group (active treatment versus placebo hf-tRNS) on the mean score will be quantified with 95%CI. The time effect will be estimated in the model by introducing a time variable at baseline, 5 days, 1 month, 3 months and 6 months after hf-tRNS, and a random effect (random slope) on the slope can be added to quantify possible heterogeneity of response and group effects on the slope of change in PANSS scores.

##### Objective 2b

We will test for a difference in secondary outcomes changes over the different levels of time between active and sham hf-tRNS using a general linear mixed-model approach. In case of significant time × treatment interaction, post hoc analyses will be conducted. Non-parametric tests will be used if the distribution is not normal. For all analyses, clinical characteristics (age, disease duration, number of previous treatments and severity of symptoms) can be introduced into the model as adjustment variables. The within-centre variability can also be estimated and taken into account in this model.

Separated statistical analysis comparing the change over time between the groups will be repeated for each of the following variables: the 5 dimensions of the PANSS score (positive, negative, depression, disorganization and grandiosity/excitement), the AHRS/HCS, the PSAS, the SNS, the CDSS, the CGI t, the S-QoL18, the SGI and the BNSS scores.

##### Objective 2c

The number of assignment errors during a source-monitoring task [33] is a countermeasure. The ANOVA comparing the mean values of the number of assignment errors before/after the intervention, and between active and sham groups will be performed using a Poisson mixed regression model with random effect on the intercept or average level. The effect of the intervention (before/after) and the effect of group (active versus placebo treatment hf-tRNS) on the mean score will be estimated with 95%CI. The within-centre variability can be estimated and included in the model.

##### Objective 3a

For each clinical parameter: age, disease duration, number of previous treatments and severity of symptoms, the proportion of responders between the active and the sham hf-tRNS groups will be estimated and compared using a Mantel-Henzel chi-square test for qualitative parameters and analysis of variance for quantitative parameters.

##### Objective 3b

Imaging data will be pre-processed and analysed according to the most recent standard procedures in the field available at the time of analysis. Focus will be made on the changes in brain activity and connectivity outcomes from baseline to post-tRNS compared between the groups (active vs sham). The brain structure, activity and connectivity at baseline will also be compared between the active and sham groups; predictive markers of responses will also be investigated using MRI data.

##### Objectives 4a and 4b

BMQ and BMQ tES scores will be compared between the active and sham groups at each time point (at baseline and at 3 months) using a Wilcoxon rank test.

The analysis of the evolution of the beliefs of patients toward medication and tES (active versus sham hf-tRNS) will quantify and test the effect of the intervention and the effect of the treatment group on the values and the slope of change in BMQ and BMQ tES scores before the intervention and after 3 months.

##### Objective 4c

A logistic regression model will be used to analyse the relationship between beliefs and clinical variables (insight, adherence), quality of life and nicotine dependence.

### Ancillary studies

The effect of the level of total serum BDNF and the relative proportions of serum BDNF isoforms before hf-tRNS on the proportion of responders will be estimated and tested using a chi-square test when the factor is categorized and an analysis of variance when the factor is analysed continuously. The effects of hf-tRNS on FAR performances will be analysed using repeated-measures ANOVA.

### Interim analyses {21b}

No interim analysis will be performed.

### Methods for additional analyses (e.g. subgroup analyses) {20b}

Subgroup analyses will be performed regarding possible confounders such as smoking status and study site. In case of unexpected baseline differences between the 2 groups (active and sham), statistical analyses could be adjusted to take into account specific variables that may have influenced the results (concurrent medication, age, sex, illness duration, etc.). Unplanned subgroups analyses by all the investigators of the study are possible after discussion with the PI, the methodologists and the steering committee of the study on reasonable request.

### Methods in analysis to handle protocol non-adherence and any statistical methods to handle missing data {20c}

To handle missing data, the analyses will be performed on a strict intention-to-treat sample of the evaluable patients defined in the protocol as patients with a baseline assessment and at least one post-tRNS score (10 sessions completed). The analyses of follow-up period will be conducted in a last observation carried forward manner through the indicated time points.

### Plans to give access to the full protocol, participant level-data and statistical code {31c}

This document is the full protocol of the study. Anonymized participant-level data and statistical code will be shared on reasonable request by scientists and may be published in a depository services website with public access after a 3-year embargo following the publication of the princeps study (clinical primary outcome).

## Oversight and monitoring

### Composition of the coordinating centre and trial steering committee {5d}

The steering committee is in charge of the trial design and study protocol, the critical review of trial-related documents, supervision of trial organization and conduct of the trial. The steering committee is composed as follow:

- Principal investigator: Prof. Emmanuel Poulet
- Methodology:
  1. Scientific advisory: Dr. Jérôme Brunelin
  2. Clinical trial methodology and study coordination: Prof. Anne-Marie Schott-Pethelaz, Dr. Julie Haesebaert and Dr. Laurent Magaud
  3. Statistical analysis: Dr. Julie Haesebaert
- Organization responsible for quality assurance and safety: Direction de la Recherche Clinique et de l’Innovation, CHU Lyon, Hospices Civils de Lyon, 3, quai des Célestins 69002 Lyon
- Organization responsible for biological central analysis: Dr. Marie-Françoise Suaud-Chagny
- Organization responsible for the imaging data: Dr. Jérôme Brunelin; Co Scientific coordinators: Prof. Renaud Jardri and Prof. Eric Fakra
- Organization responsible for data management: Dr. Laurent Magaud

### Composition of the data monitoring committee, its role and reporting structure {21a}

Data monitoring will be ensured by the sponsor of the study without any relationship with the investigators. Onsite monitoring visits are planned throughout the study period: first monitoring after the first inclusions and then at least once per year.

### Adverse event reporting and harms {22}

Safety assessment will be conducted according to article R1123-46 of the Public Health Code, MDR 2017/745 and MDCG 2020-10/1, France.

The relationship between the use of the medical device and the occurrence of each adverse event will be assessed and categorized.

#### Adverse event (AE), serious or not reporting

For each patient participating in the trial, the investigator will document, in the AE section of the case report form (CRF), any AE (serious or not) and device deficiency that occurs from the start of screening until the end-of-study examination. This will include any AE that the investigator observed as well as any AE that a patient reported spontaneously or in response to a non-leading question. The AE documentation includes AE dates (start and end), outcome, measures taken, the causality and intensity.

#### Follow-up of adverse events and serious adverse events

All adverse events must be documented and the outcome must be followed up until they return to normal or consolidation of the patient’s condition. Any SAE should be monitored until they are resolved or stable. The investigator is responsible for following the SAE evolution until the event is completely resolved. Any relevant information concerning the SAE that becomes available should be forwarded as soon as possible to the sponsor.

### Sponsor responsibility

According to article R1123-55 of the Public Health Code, MDR 2017/745 and to the Medical Device Coordination Group (MDCG) guidelines 2020-10/1, the sponsor shall report to the competent authorities any reportable events.

### Frequency and plans for auditing trial conduct {23}

By signing the protocol, the investigator agrees to allow the sponsor and its representative and regulatory agencies to have direct access to his study records for a site audit or an inspection. These personnel, bound by professional secrecy, will not disclose any personal identity or personal medical information.

In all cases, the sponsor will help the investigator prepare for an inspection by any regulatory authority.

### Plans for communicating important protocol amendments to relevant parties (e.g. trial participants, ethical committees) {25}

According to national regulations, major protocol modifications require a formal amendment to the protocol and have to be approved by the IRB. The study sponsor will be in charge of communicating protocol modifications to participating study sites.

### Dissemination plans {31a}

Several international publications and communications during national and international congresses are planned. The primary outcome clinical measure will be published in the first article. The results from secondary outcomes and ancillary studies could be published in separate articles. All the articles from the Stim’Zo study database must refer to the PRINCEPS published study.

Anonymized participant-level data may be published in a depository services website with public access after a 3-year embargo following the publication of the PRINCEPS study (clinical primary outcome).

## Discussion

The present trial is a double-blind, sham-controlled, parallel-group trial testing the efficacy of high-frequency transcranial random noise stimulation (hf-tRNS) as an add-on treatment in patients with schizophrenia. This trial will constitute a first step toward establishing the feasibility and efficacy of this technique for the treatment of resistant/persistent symptoms of schizophrenia in patients with various profiles of symptoms, cognitive deficits and illness duration.

While several RCTs have already been conducted to test the efficacy of tDCS in schizophrenia, most of them were limited by the small sample size and lack of follow-up assessment. Here, besides proposing a new tDCS protocol by delivering hf-tRNS instead of direct constant current, we aim to conduct an RCT with a large sample size and follow-up assessments up to 6 months. This will help investigate the long-lasting effect of this technique. We proposed to deliver a 10-session regimen as previously reported with promising clinical benefits in patients with schizophrenia [11, 12]. However, recent studies tend to increase the number of sessions to improve the clinical efficacy [73], but see [74] for negative results; further studies will be developed to assess the clinical interest of increasing the number of sessions during the acute phase treatment as well as to propose maintenance protocol after clinical response. This study was not designed to investigate these points of interest. We believe that establishing the acute beneficial effects of hf-tRNS is mandatory first before developing maintenance protocols.

From a clinical perspective, one limitation that is commonly raised toward noninvasive brain stimulation techniques is the heterogeneity of clinical response to these techniques. By investigating a wide range of biological, cognitive and clinical outcomes, the current RCT will contribute to identifying some predictors of clinical response. From a research perspective, this investigation of biological, cognitive and clinical outcomes over time will help to understand the mechanisms by which hf-tRNS act to reduce symptoms in schizophrenia. As some subtle disabling symptoms and cognitive deficits are considered as core features of schizophrenia, as well as good predictive vulnerability markers of the illness, we also hypothesize that hf-tRNS would be clinically relevant in at-risk subjects and therefore could constitute a preventive intervention against psychotic onset in at-risk subjects in the future.

Overall, if the findings from the present RCT are positive, they may contribute to placing hf-tRNS as a significant option for the treatment of treatment-resistant symptoms of schizophrenia.

## Trial status

The current protocol version is 13 (date July 13, 2021). Recruitment began in 2016 and is expected to end in 2022.

## Abbreviations

AE: Adverse event
AHRS: Auditory Hallucination Rating Scale
ASL: Arterial spin labelling
BDNF: Brain-derived neurotrophic factor
BMQ: Beliefs about Medicines Questionnaire
BNSS: Brief Negative Symptom Scale
BOLD: Blood oxygen level-dependent
CDSS: Calgary Depression Scale for Schizophrenia
CGI: Clinical Global Impressions
CRF: Case report form
DSM: Diagnostic and Statistical Manual of Mental Disorders
DTI: Diffusion tensor imaging
eCRF: Electronic case report form
FAR: Facial affect recognition
fMRI: Functional magnetic resonance imaging
FTND: Fagerstrom test for nicotine dependence
HCS: Hallucination changes scores
hf-tRNS: High-frequency transcranial random noise stimulation
MARS: Medication Adherence Rating Scale
MRI: Magnetic resonance imaging
PANSS: Positive and Negative Syndrome Scale
PSAS: Psycho-Sensory hAllucinations Scale
RCT: Randomized controlled trial
rTMS: Repetitive transcranial magnetic stimulation
SAE: Serious adverse event
SGI: Sensory Gating Inventory
SNS: Self-evaluation of negative symptoms
SUMD: Scale to Assess Unawareness of Mental Disorder
S-QoL18: Schizophrenia Quality of Life Questionnaire Short Form
tDCS: Transcranial direct current stimulation
tES: Transcranial electrical stimulation
tRNS: Transcranial random noise stimulation
VAS: Visual analogue scale
